# High proportion of circulating CD8 + CD28- senescent T cells is an independent predictor of distant metastasis in nasopharyngeal canrcinoma after radiotherapy

**DOI:** 10.1186/s12967-023-03912-2

**Published:** 2023-01-31

**Authors:** Xiaotian Xu, Fangze Wei, Lin Xiao, Runye Wu, Baojun Wei, Shengkai Huang, Junlin Yi, Wei Cui

**Affiliations:** 1 Department of Clinical Laboratory, State Key Laboratory of Molecular Oncology, National Cancer Center/National Clinical Research Center for Cancer/Cancer Hospital, Chinese Academy of Medical Sciences and Peking Union Medical College, Beijing, 100021 China; 2 Department of Radiation Oncology, National Cancer Center/National Clinical Research Center for Cancer/Cancer Hospital, Chinese Academy of Medical Sciences and Peking Union Medical College, Beijing, 100021 China

**Keywords:** Nasopharyngeal carcinoma, Radiotherapy, Prognosis, CD8^+^CD28^−^ T cell, Immune senescence

## Abstract

**Background:**

Nasopharyngeal carcinoma (NPC) is a kind of epithelial carcinoma that is common in East and Southeast Asia. Distant metastasis after radiotherapy remains the main cause of treatment failure and preradiotherapy immune system function can influence prognosis. Our study aimed to identify immune-related prognostic factors for NPC after radiotherapy and establish a prognostic model to predict progression-free survival (PFS) and distant metastasis-free survival (DMFS).

**Methods:**

We enrolled NPC patients and divided them into training and validation cohorts with follow-up. We collected clinical information and investigated immune cells, EBV DNA and cytokines in the peripheral blood of NPC patients before radiotherapy and EBV DNA after radiotherapy. Among these immune cells, we included CD8^+^CD28^−^ T cells, which are a unique T-cell immunosenescent subset that increases in human peripheral blood with increasing age and declining immune function. Based on the detection results and clinical information, we utilized Cox regression and least absolute shrinkage and selection operator (LASSO) regression to screen the PFS and DMFS prognostic factors and build nomograms to predict the PFS and DMFS of NPC. We also verified the results in the validation set.

**Results:**

Three factors associated with PFS were selected: proportion of CD8^+^CD28^−^ T cells posttreatment EBV and N stage. Three factors associated with DMFS were screened: proportion of CD8^+^CD28^−^ T cells, posttreatment EBV and N stage. CD8^+^CD28^−^ T cells are correlated with systemic inflammation and posttreatment immunosuppression. The C-indexes were 0.735 and 0.745 in the training and validation cohorts for predicting PFS. For DMFS, the C-indexes were 0.793 and 0.774 in the training and validation cohorts.

**Conclusions:**

The pretreatment proportion of CD8^+^CD28^−^ T cells is a candidate prognostic biomarker for NPC after radiotherapy. The constructed nomogram models based on CD8^+^CD28^−^ T cells have good predictive value.

**Supplementary Information:**

The online version contains supplementary material available at 10.1186/s12967-023-03912-2.

## Background

Radiotherapy is the mainstay treatment modality for nonmetastatic nasopharyngeal carcinoma (NPC) because of its high sensitivity to ionizing radiation [1]. Although the extensive use of intensity-modulated radiotherapy (IMRT) and concurrent chemoradiotherapy (CCRT) improves the treatment efficacy and reduces the mortality of NPC, distant metastasis after radiotherapy is still the main treatment challenge, and the curative effect of salvage treatment after recurrence remains unsatisfactory [2, 3]. The current anatomic staging system is not sufficient for predicting the prognosis or therapeutic effect. Therefore, it is important to screen and identify biomarkers related to the stratification of prognostic risk and treatment benefit.

Radiotherapy has a dual function in immune system regulation: it has an immunostimulatory effect and an immunosuppressive effect. Therefore, the status of immune function is very important. Activation of the antitumor-specific immune response induced by radiotherapy plays an important role in the systemic control and prognosis improvement of tumors [4]. In addition, radiotherapy can induce cancer cell death by mediating DNA damage and regulating both immune-related mediators and antigens [5]. Recent studies in immunotherapy have further confirmed the synergistic effect of radiotherapy and immune checkpoint inhibitors on tumor immune response activation [6, 7]. Therefore, the maintenance, recovery and continuous activation of antitumor-specific immune function is the key to achieving therapeutic effects, regardless of whether traditional chemotherapy and radiotherapy or immunotherapy are used.

In some animal models and preclinical studies, the abscopal effect [8] can induce continuous systemic antitumor immune activation and plays an important role in controlling metastasis. However, in clinical practice, radiotherapy has both immunostimulatory and immunosuppressive effects underlying the complicated immune landscape in patients receiving radiotherapy [9]. Consequently, it is essential to assess the immune system at the patient level to optimize and monitor individualized treatment approaches. Lymphocyte subsets are important tools for detecting the immune status of the body. In our study, we detected peripheral blood lymphocyte subsets simultaneously with plasma Epstein‒Barr virus (EBV) DNA and cytokines in patients with NPC and screened key factors associated with early recurrence and distant metastasis after radiotherapy. The results showed that pretreatment proportion of CD8^+^CD28^−^ T cells in CD8 T cell can serve as a new prognostic biomarker for NPC after radiotherapy, and the nomograms we developed have good predictive value for progression-free survival (PFS) and distant metastasis-free survival (DMFS).

## Methods

### Patients and follow-up

Our study used the following criteria to screen patients for inclusion: newly diagnosed stage I-IVa NPC; did not receive any antitumor therapy before biopsy sampling; received radical IMRT with or without chemotherapy; age  ≥ 18 years; Eastern Cooperative Oncology Group (ECOG) score between 0 and 2; adequate hematological, renal, and hepatic functions; and no malignant disease.

We analyzed a total of 332 previously untreated patients with biopsy-proven NPC and no evidence of distant metastasis (M0) screened by fiberoptic nasopharyngoscopy and imageological examination at Cancer Hospital from June 2014 to April 2018. We collected patients’ basic clinical information and explored peripheral blood immune cell profile. The follow-up clinical data were reviewed in the clinical system of our hospital or obtained by telephone and email. DMFS was defined as the interval between the date of diagnosis and the advent of the first distant metastasis event, and PFS was defined as the interval between the date of diagnosis and the date of disease progression or death from any cause. In the end, some patients were lost to follow-up. In screening prognostic factors, we included 293 samples and divided the patients into training and validation groups. For the training cohort, 131 samples were obtained from patients between June 20, 2014, and March 16, 2015, and for the validation cohort, 162 samples were obtained between September 12, 2015, and April 6, 2018. In addition, 176 healthy volunteers were recruited to serve as controls. This study was approved by the Cancer Hospital. The clinical information and characteristics were recorded after consent was obtained from all participants of this study.

### Flow cytometric analysis

Fresh blood samples were collected from patients and volunteers. Six-color flow cytometric analysis was performed to determine cell phenotypes. Nine specific monoclonal antibodies (BD Biosciences, San Jose, CA, USA) against CD3 (FITC), CD45 (Per CP), CD4 (FITC and APC), CD45RO (PE), CD8 (PE-CY7), CD19 (APC), CD28 (PE), CD56 (PE), and HLA-DR (APC) were used to differentiate lymphocyte subsets. Lymphocytes were gated by CD45. The lymphocyte subsets analyzed included T cells (CD3^+^, CD3^+^CD4^+^, CD3^+^CD8^+^, CD4^+^CD45RO^+^, CD8^+^CD28^+^, CD8^+^CD28^−^, and CD3^+^HLA-DR^+^), natural killer cells (CD3^−^CD56^+^), and B cells (CD3^−^CD19^+^). Flow cytometry was performed using a BD Canto II system, and BD Diva software was used for data analysis.

### Extraction of EBV DNA from plasma

A total of 200 ml per column of the plasma sample was used for DNA extraction; plasma samples were collected from the patients as described and stored at − 80 °C. We extracted plasma sample DNA utilizing a QIAamp blood kit (Qiagen, Hilden, Germany) with the blood and body fluid protocol. Levels of circulating EBV DNA were measured with real-time quantitative PCR (qPCR), which amplified a DNA segment in the EBNA1 region of the EBV genome. The principles of real-time quantitative PCR and reaction setup procedures were as described. The data were collected with an ABI PRISM 7500 sequence detector (Applied Biosystems, Foster City, CA) and analyzed with Sequence Detection System software (version 1.6.3) developed by Applied Biosystems. The results are expressed as the number of copies of EBV genomes per milliliter of plasma.

### Plasma cytokine assays

EDTA‐K2 anti‐coagulated whole blood was centrifuged at 1000 × g for 30 min, and then plasma samples were harvested and stored at − 80 °C until further processing. The concentrations of interleukin 1β (IL-1β), IL-2, IL-4, IL-5, IL-6, IL-8, IL-10, IL-12p70, IL-17, IFN-γ, IFN-α and TNF-α in plasma samples were measured by multiple microsphere flow immunofluorescence using commercially available kits (Raisecare Biotechnology Co., Ltd., Shandong, China; lot number: 20190801) according to the manufacturer’s instructions. A total of 25 μl of experimental buffer, 25 μl of centrifuged plasma, 25 μl of capture microsphere antibody, and 25 μl of detection antibody were added to the corresponding flow tubes. After incubation at room temperature for 2 h in darkness with gentle shaking, 25 μl of streptavidin‐phycoerythrin (SA‐PE) was added to the flow tubes, and incubation was continued for an additional 30 min. Subsequently, diluted wash buffer (1:10) was added. After a few seconds of vortex shaking, the flow tube was centrifuged at 432 × g for 5 min. The liquid was slowly poured out, and the flow tube was inverted on absorbent paper. Then, 100 μl of diluted washing buffer (1:10) was added to the flow tube, which was shaken for 10 s followed by detection.

### Statistical analysis

We utilized univariate Cox (uniCox) regression analysis, least absolute shrinkage and selection operator (LASSO) and multivariate Cox (multiCox) regression analysis to screen the factors of PFS and DMFS in the training cohort and then used the validation cohort to validate the factors. Based on the key prognostic factors selected by analysis, we established nomograms utilizing the R package “rms” and used calibration curves and the C-index to validate the predictive value of the nomograms. Other statistical analyses were performed in SPSS (RRID: SCR_002865) and GraphPad Prism (RRID: SCR_002798). All statistical tests were two-sided, and P values of less than 0.05 were deemed significant.

## Results

### Characteristics and clinical outcome of the patients

**Fig. 1:**
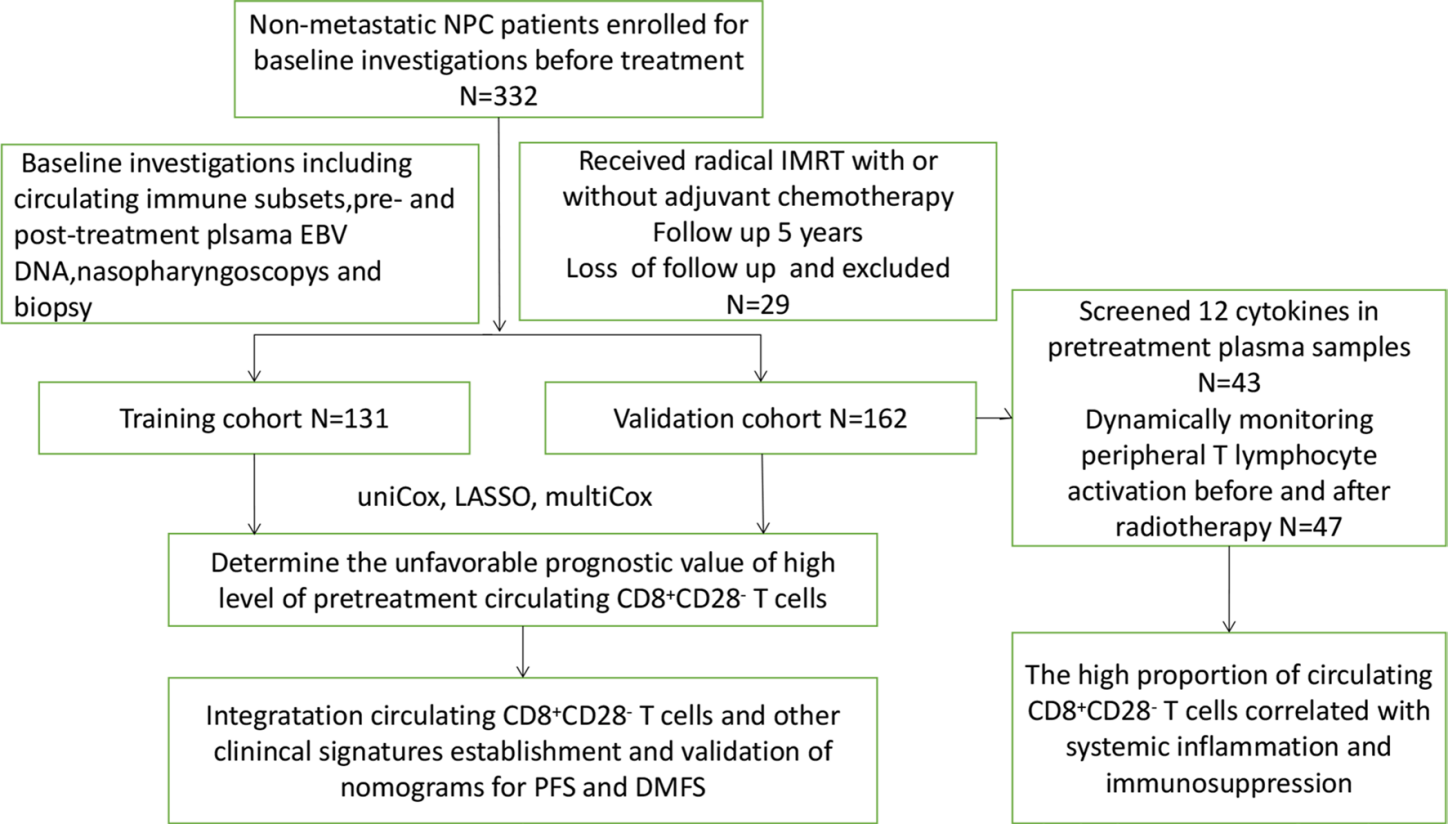
Schematic diagram. Radiotherapy can influence immune function. We studied nasopharyngeal cancer patients’ immune function after radiotherapy and identified immune-related prognostic factors associated with distant metastasis utilizing a mathematical method. The nomogram based on the prognostic markers had good predictive value for DFS and DMFS. We found that CD8^+^CD28^−^ T cells are associated with stage, EBV counts, radiotherapy treatment and age and can be a potential prognostic biomarker for nasopharyngeal cancer patients who receive radiotherapy

A schematic diagram of the study is shown in Fig. 1. The raw data before and after follow-up are provided in Additional file 1 and Additional file 2. As shown in Table 1, basic clinical information included age, sex, pathological type according to the World Health Organization, response to chemotherapy, T stage, N stage, clinical stage, pretreatment plasma EBV DNA concentration and pretreatment immune cells profile including proportions of CD8^+^CD28^−^ T cells and other kinds of immune cells; we also included plasma EBV DNA one week after the completion of treatment. We divided the patients into two groups based on the median of CD8^+^CD28^−^ T cells proportions. The patient characteristics of the training and validation cohorts are shown in Table 1 and Additional file 3. A total of 96 (73.3%) patients had no progression, and 35 (26.7%) patients had progression; 112 (85.5%) patients had no distant metastasis, and 19 (14.5%) patients had distant metastasis. For the validation cohort, 127 (78.4%) patients were men, and 35 (21.6%) patients were women; the median age was 47 years (range: 15–73). A total of 119 (73.5%) patients had no progression, and 43 (26.5%) patients had progression; 130 (80.2%) patients had no distant metastasis, and 32 (19.8%) patients had distant metastasis.

**Table 1:** Characteristics of the patients

Variable		Training (131)	%	Validation (162)		P
Sex	1	105	80.2	127	78.4	0.713
	2	26	19.8	35	21.6	
Age	Median	48		47		0.611
	Range	21–80		15–73		
EBV	Undetectable	54	41.2	37	22.8	0.003
	< 1500 copies/ml	53	40.5	91	56.2	
	> 1500 copies/ml	24	18.3	34	21	
Posttreatment EBV	Undetectable	128	97.7	149	92	0.032
	Detectable	3	2.3	13	8	
T	T1	13	9.9	11	6.8	0.582
	T2	16	12.2	26	16	
	T3	73	55.7	85	52.5	
	T4	29	22.1	40	24.7	
N	N0	12	9.2	5	3.1	0.008
	N1	49	37.4	41	25.3	
	N2	47	35.7	80	49.4	
	N3	23	17.6	36	22.2	
Stage	I	1	0.8	2	1.2	0.243
	II	15	11.5	11	6.8	
	III	72	55	80	49.4	
	IV	43	32.8	69	42.6	

### Key prognostic factor selection for PFS and DMFS

Based on the data of lymphocyte subsets and clinical signatures, we performed uniCox, LASSO and multiCox regression analyses to screen the factors for PFS and DMFS (Additional file 4 and Additional file 5).

**Fig. 2:**
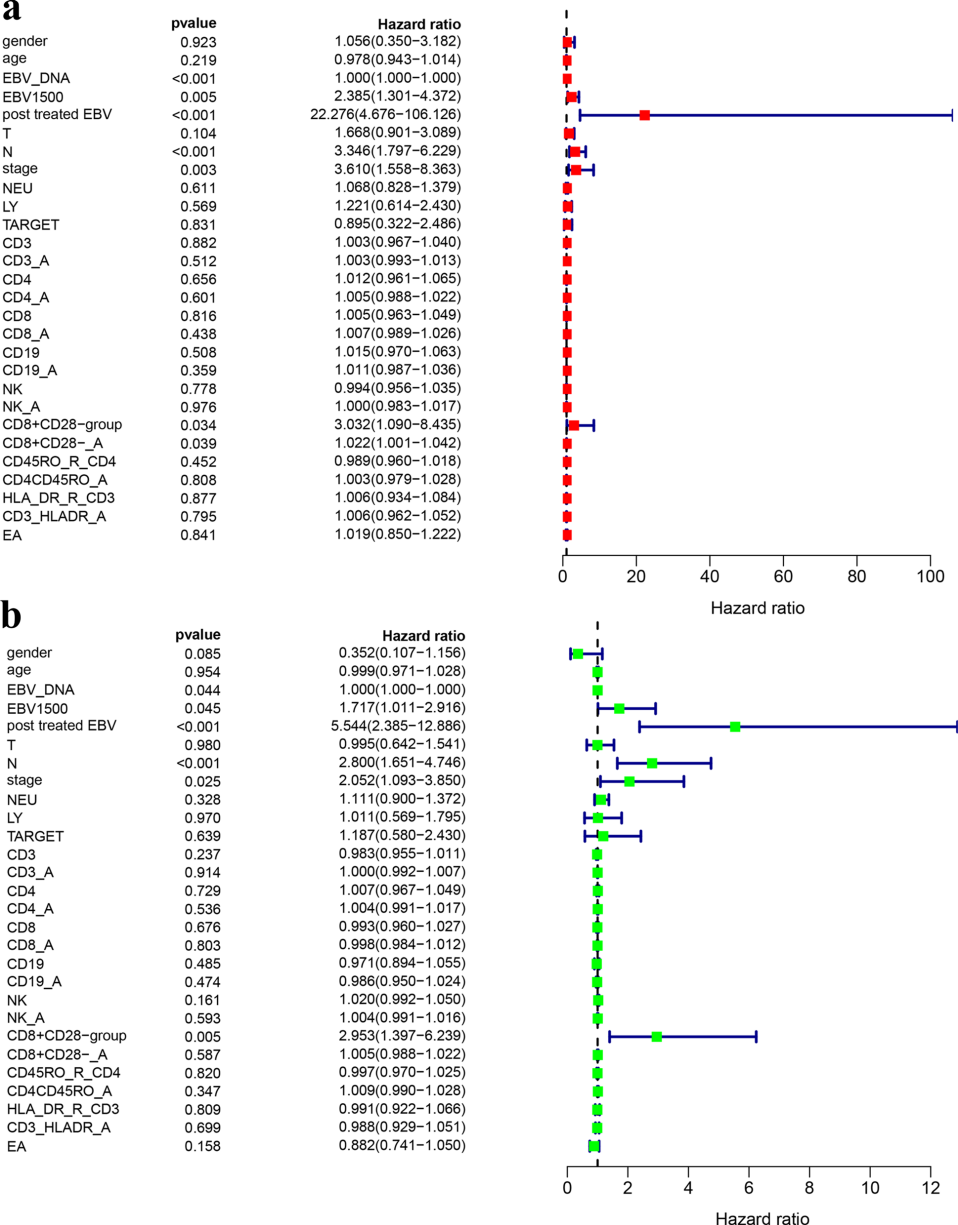
UniCox regression analysis of predicting DMFS in the training and validation cohorts. **a** UniCox forest map in the training cohort, and the red diamonds represent the median expression. **b** UniCox forest map in the validation cohort, and the green diamonds represent the median expression. The blue line represents the 95% CI. CD3: proportion of CD3 T cells (%); CD3_A: absolute count of CD3 T cells (cells/μl); CD4: proportion of CD4 T cells (%); CD4_A: absolute count of CD3 T cells (cells/μl); CD8: proportion of CD8 T cells (%); CD8_A: absolute count of CD8 T cells (cells/μl); CD19: proportion of CD3^−^CD19^+^ B cells (%); CD19_A: absolute count of CD3^−^CD19^+^ B cells (cells/ul); NK: proportion of CD3^−^CD56^+^ NK cells (%); NK_A: absolute count of CD3^−^CD56^+^ NK cells (cells/μl); CD8^+^CD28^−^ group: relative proportion of CD28neg senescent T cells in CD8 T cells; CD8^+^CD28^−^_A: absolute count of CD8^+^CD28^−^_Tcells (cells/μl); CD45R0_R_CD4: relative proportion of CD45R0^+^ in_CD4 T cells; CD4CD45R0_A: absolute count of CD4^+^CD45R0^+^ T cells (cells/μl); HLA_DR_R_CD3: relative proportion of HLA-DR^+^ in CD3 T cells; CD3_HLADR_A: absolute count of CD3^+^HLADR^+^ T cells (cells/μl); EA: EA-IgA EB viral early antigen IgA antibody

**Fig. 3:**
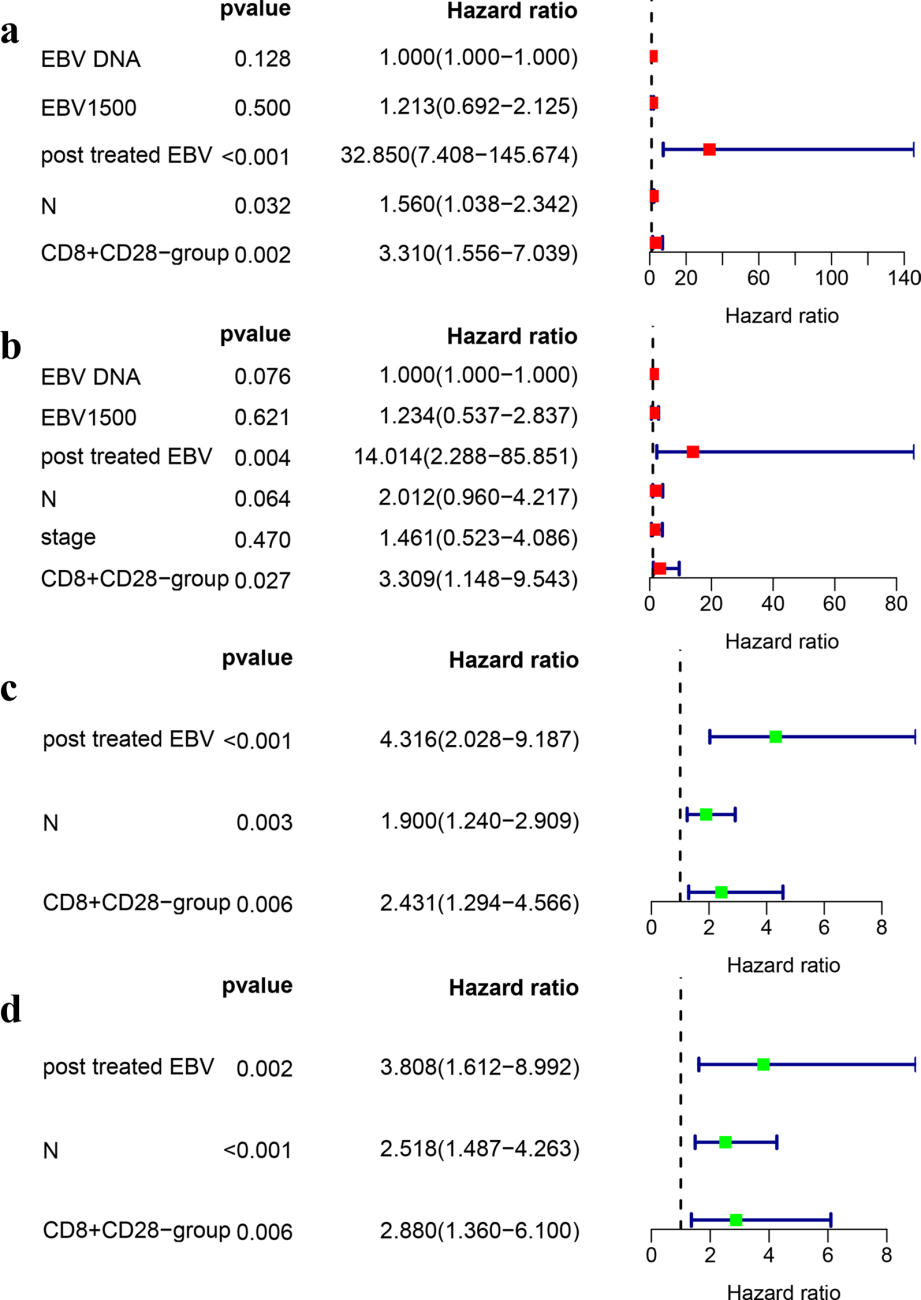
MultiCox regression analysis in the training and validation cohorts for predicting PFS and DMFS. **a** PFS multiCox forest map in the training cohort. **b** DMFS multiCox forest map in the training cohort. **c** PFS multiCox forest map in the validation cohort. **d** DMFS multiCox forest map in the validation cohort. The diamonds represent the median expression and the blue line represents the 95% CI

For PFS, in the training cohort, 6 potential prognostic factors were selected by uniCox analysis: proportion of CD8^+^CD28^−^ T cells (P = 0.003) and CD8^+^CD28^−^ absolute count (P = 0.033); clinical information: N stage (P = 0.001), copies of EBV DNA in plasma (P = 0.005), EBV DNA copies more than 1500 (P = 0.01) and posttreatment EBV status (P < 0.001). In the validation cohort, 4 potential prognostic factors were selected by uniCox analysis: proportion of CD8^+^CD28^−^ T cells (P = 0.004), posttreatment EBV status (P < 0.001), N stage (P < 0.001) and clinical stage (P = 0.017). For LASSO regression, in the training cohort, 5 potential factors were selected:proportion of CD8^+^CD28^−^ T cells; clinical information: posttreatment EBV status, N stage, copies of EBV DNA and EBV DNA copies more than 1500. In the validation cohort, 3 potential factors were selected by LASSO regression: proportion of CD8^+^CD28^−^ T cells; clinical information: posttreatment EBV status and N stage. Then, in multiCox analysis, 3 prognostic factors were selected in the training cohort: proportion of CD8^+^CD28^−^ T cells (P = 0.002), posttreatment EBV status (P < 0.001) and N stage (P = 0.032) (Fig. 3a); in the validation cohort, 3 prognostic factors were selected: proportion of CD8^+^CD28^−^ T cells (P = 0.006), posttreatment EBV (P < 0.001) and N stage (P = 0.003) (Fig. 3c). For DMFS, forest plots are shown in Figs. 2, in the training cohort, 7 potential prognostic factors were selected by uniCox analysis: proportion of CD8^+^CD28^−^ T cells (P = 0.034), CD8^+^CD28^−^ absolute count (P = 0.039), posttreatment EBV status (P < 0.001), N stage (P < 0.001), copies of EBV DNA in plasma (P < 0.001), EBV DNA copies more than 1500 (P = 0.005) and stage (P = 0.003) (Fig. 2a). In the validation cohort, 6 prognostic factors were selected: proportion of CD8^+^CD28^−^ T cells (P = 0.005), posttreatment EBV (P < 0.001), N stage (P < 0.001), stage (P = 0.025), copies of EBV DNA in plasma (P = 0.044) and EBV DNA copies more than 1500 (P = 0.045) (Fig. 2b). For LASSO regression, 6 potential factors were selected in the training cohort: proportion of CD8^+^CD28^−^ T cells, posttreatment EBV status, N stage, copies of EBV DNA in plasma, stage and EBV DNA copies more than 1500. In the validation cohort, 3 factors were selected: proportion of CD8^+^CD28^−^ T cells, posttreatment EBV status and N stage. Two prognostic factors were selected through multiCox analysis: proportion of CD8^+^CD28^−^ T cells (P = 0.026) and posttreatment EBV status (P = 0.004) in training cohort (Fig. 3b). In the validation cohort, 3 factors were selected: proportion of CD8^+^CD28^−^ T cells (P = 0.006), posttreatment EBV status (P = 0.002), and N stage (P < 0.001) (Fig. 3d).

### ***Circulating CD8***^+^***CD28***^***−***^*** T cells in patients with NPC***

**Fig. 4:**
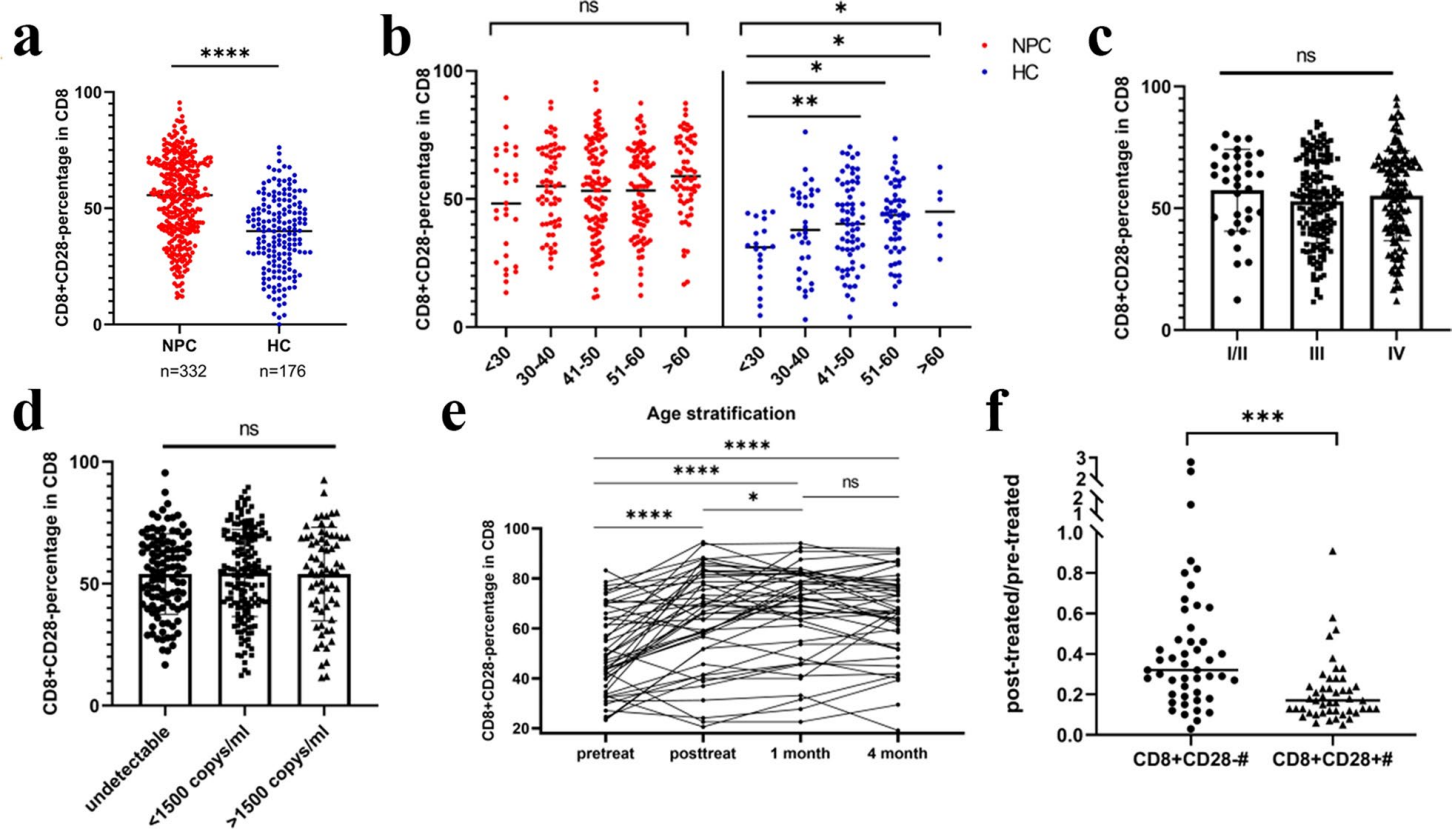
Clinical analyses of circulating CD8^+^CD28^−^ T cells in patients with nasopharyngeal carcinoma and controls. **a** The proportion of circulating CD8^+^CD28^−^ T cells in patients with nasopharyngeal carcinoma and healthy volunteers. **b** CD8^+^CD28^−^ T-cell proportion in different age stratifications. **c** The pretreatment proportion of circulating CD8^+^CD28^−^ T cells in patients with nasopharyngeal carcinoma was not correlated with the clinical stage of disease. **d** The CD8^+^CD28^−^ percentage in CD8 T cells with the plasma EBV DNA concentration. **e** Radiotherapy further increased the proportion of CD8^+^CD28^−^ T cells in peripheral blood, and the increase lasted 4 months after treatment. **f** CD8^+^CD28^−^ T cells are insensitive to the cell death induced by radiotherapy. At the end of radiotherapy, the absolute count of CD8^+^CD28^+^ T cells in peripheral blood decreased much more than that of CD8^+^CD28^−^ T cells. *: P < 0.05; **: P < 0.01; ***: P < 0.001; ****: P < 0.0001

The percentage of CD8^+^CD28^−^ T cells in CD8^+^ T cells in the peripheral blood of NPC patients was significantly higher than that in the healthy controls (P < 0.0001, Fig. 4a). The raw data of healthy controls are shown in Additional file 6. The increased CD8^+^CD28^−^ T cell subsets in the peripheral blood of NPC patients dont’t have relationship with age, clinical stage or plasma EBV DNA concentration (Fig. 4b–d).

Dynamic monitoring of peripheral blood lymphocyte subsets before and after radiotherapy (n = 47) showed that after radiotherapy, the proportion of CD8^+^CD28^−^ T cells in peripheral blood was higher than that before radiotherapy (P < 0.0001). After radiotherapy for 1 month, the proportion of CD8^+^CD28^−^ T cells was higher than that before radiotherapy (P < 0.001). After radiotherapy for 4 months, the proportion of CD8^+^CD28^−^ T cells was higher than that before radiotherapy (P < 0.0001) (Fig. 4e). Comparing the absolute count of each subgroup at the end of radiotherapy,CD8^+^CD28^−^ T cells were reduced less than CD8^+^CD28^+^ T cells (P < 0.001) (Fig. 4f).

### *The correlations between CD8* + *CD28- T cells and systemic inflammation and posttreatment immunosuppression*

**Fig. 5:**
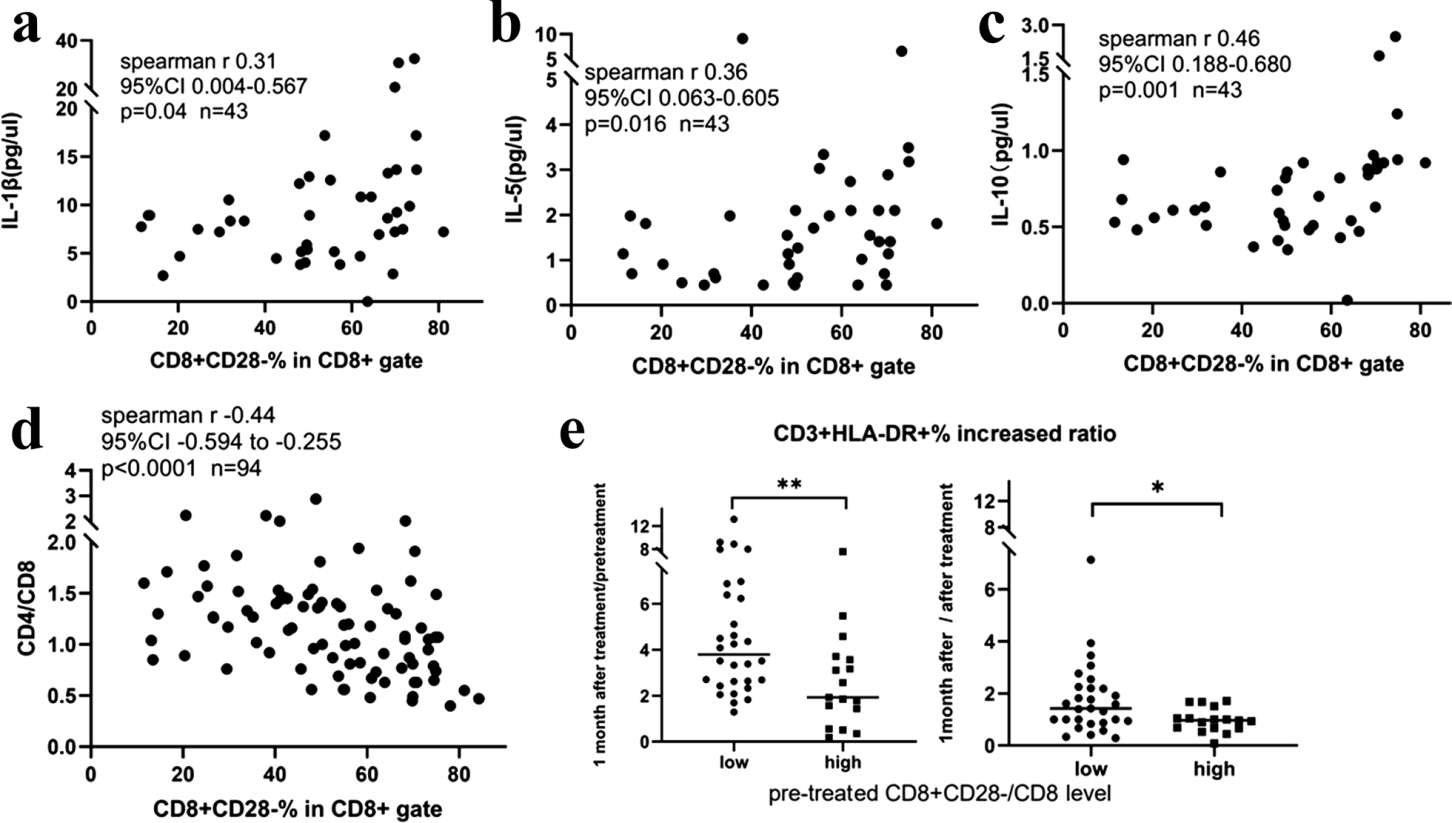
Association of CD8^+^CD28^−^ T cell proportion with systemic immune biomarkers in blood. **a** Correlation scatter plot between IL-1 and the CD8^+^CD28^−^ T cell proportion. **b** Correlation scatter plot between IL-5 and the CD8^+^CD28^−^ T cell proportion. **c** Correlation scatter plot between IL-10 and the CD8^+^CD28^−^ T cell proportion. **d** Correlation scatter plot between CD8^+^CD28^−^ T cell proportions. **e** In the group with a high level of pretreatment CD8^+^CD28^−^ T cells in peripheral blood, the increase in CD3^+^HLA-DR^+^ activated T cells after radiotherapy was significantly lower than that in the group with a low level of CD8^+^CD28^−^ T cells

In the validation cohort, we screened 12 cytokines in pretreatment plasma samples from 43 patients, and the results are shown in Additional file 7. The results showed that peripheral blood CD8^+^CD28^−^ T cells were positively correlated with the inflammatory cytokines IL-1β and IL-5 and the immunosuppressive cytokine IL-10 (Fig. 5a–c). The proportion of CD8^+^CD28^−^ T cells in CD8 T cells was inversely proportional to CD4/CD8 (n = 94, P < 0.0001) (Fig. 5d). Dynamically monitoring 47 patients with peripheral blood lymphocyte subsets before and after radiotherapy, the number of CD3^+^HLA-DR^+^ activated T cells was also detected. In the group with high levels of pretreatment CD8^+^CD28^−^ T cells, the increase in CD3^+^HLA-DR^+^ activated T cells after radiotherapy for one month was significantly lower than that in the group with low levels of CD8^+^CD28^−^ T cells (Fig. 5e).

### Prognostic value of the CD28 expression level on CD8 T cells

**Fig. 6:**
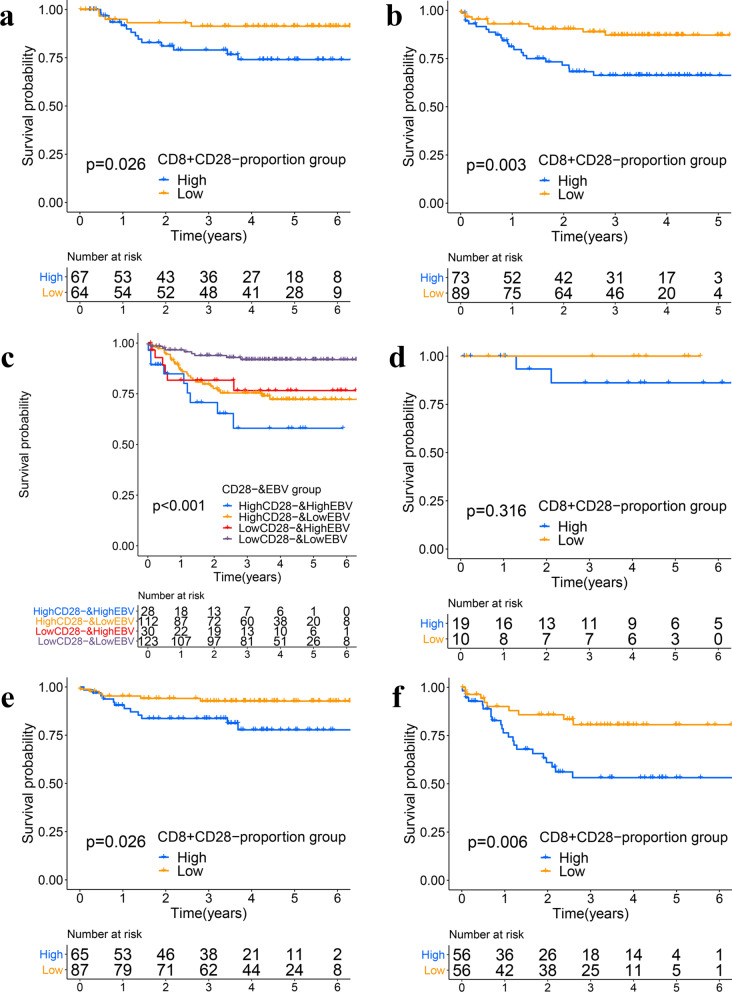
Kaplan-Meier plot for CD28^+^CD28^−^ T-cell and clinical information relationship. **a** Kaplan-Meier plot for the proportion of CD8^+^CD28^−^ T cells for DMFS in the training cohort. **b** Kaplan-Meier plot for the proportion of CD8^+^CD28^−^ T cells for DMFS in the validation cohort. **c** Kaplan-Meier plot for CD8^+^CD28^−^ T cells combined with EBV DNA for DMFS. The purple line represents a low proportion of CD8^+^CD28^−^ T cells and EBV < 1500; the yellow line represents a high proportion of CD8^+^CD28^−^ T cells and EBC < 1500; the red line represents a low proportion of CD8^+^CD28^−^ T cells and EBC > 1500; and the blue line represents a high proportion of CD8^+^CD28^−^ T cells and EBC > 1500. **d** Kaplan-Meier plot for the proportions of CD8^+^CD28^−^ T cells in stage I/II patients. **e** Kaplan-Meier plot for the proportions of CD8^+^CD28^−^ T cells in stage III patients. **f** Kaplan-Meier plot for the proportions of CD8^+^CD28^−^ T cells in stage IV patients. The yellow line represents a low proportion of CD8^+^CD28^−^ T cells, and the blue line represents a high proportion of CD28^+^CD28^−^ T cells

Kaplan‒Meier estimates showed that pretreatment circulating CD8^+^CD28^−^ T cells were significantly correlated with DMFS and PFS. In the training cohort, high levels (> 56%) of pretreatment peripheral CD8^+^CD28^−^ T cells were closely associated with PFS (P = 0.002) (Additional file 8a) and DMFS (P = 0.026) (Fig. 6a). In the validation cohort, high levels (> 56%) of pretreatment peripheral CD8^+^CD28^−^ T cells were closely associated with PFS (P = 0.003) (Additional file 8b) and DMFS (P = 0.003) (Fig. 6b).

Lin et al. [10] demonstrated that EBV DNA levels are associated with NPC prognosis, and the cutoff point for EBV DNA expression is 1500 copies/ml. Previous studies showed that plasma EBV DNA levels were considered complementary factors for TNM stage [11]. However, in our study, patients with pretreatment plasma EBV DNA levels lower than 1500 copies/ml (n = 235) still experienced recurrence (n = 78) or distant metastasis (n = 51) during follow-up. Therefore, we further analyzed the prognostic value of CD8^+^CD28^−^ T cells in patients with different levels of plasma EBV DNA. As shown in Fig. 6c, in patients with pretreatment plasma EBV DNA below 1500 copies/ml, those with a high proportion of CD8^+^CD28^−^ T cells had a significantly higher risk of distant metastasis after radiotherapy. However, in patients with pretreatment plasma EBV DNA above 1500 copies/ml, the proportion of CD28^−^ CD8 T cells have a prognostic tendency butdid not reach statistical significance for the prediction of recurrence (P < 0.001) (High EBVgroups: High VS Low proportion of CD8 + CD28-T cells P = 0.216; Low EBV groups: High VS Low proportion of CD8 + CD28- T cells is P < 0.001).

Previous data have demonstrated that anatomical staging is not sufficient for distinguishing the prognosis between stage II and III patients [11]. This is consistent with the data in this study as shown in Additional file 9. We further analyzed the prognostic value of pretreatment CD28^−^ CD8 T cells in patients with different stages and found that CD28^−^ CD8 T cells have definite prognostic value for distant metastasis after radiotherapy in patients with different clinical stages (Fig. 6d–f). Specifically, patients with a proportion of CD8^+^CD28^−^ < 56% have a better prognosis and have prognostic tendency (for stage I/II, P = 0.316; for stage III, P = 0.026; and for stage IV, P = 0.006).

### Establishment and validation of nomograms for PFS and DMFS

**Fig. 7:**
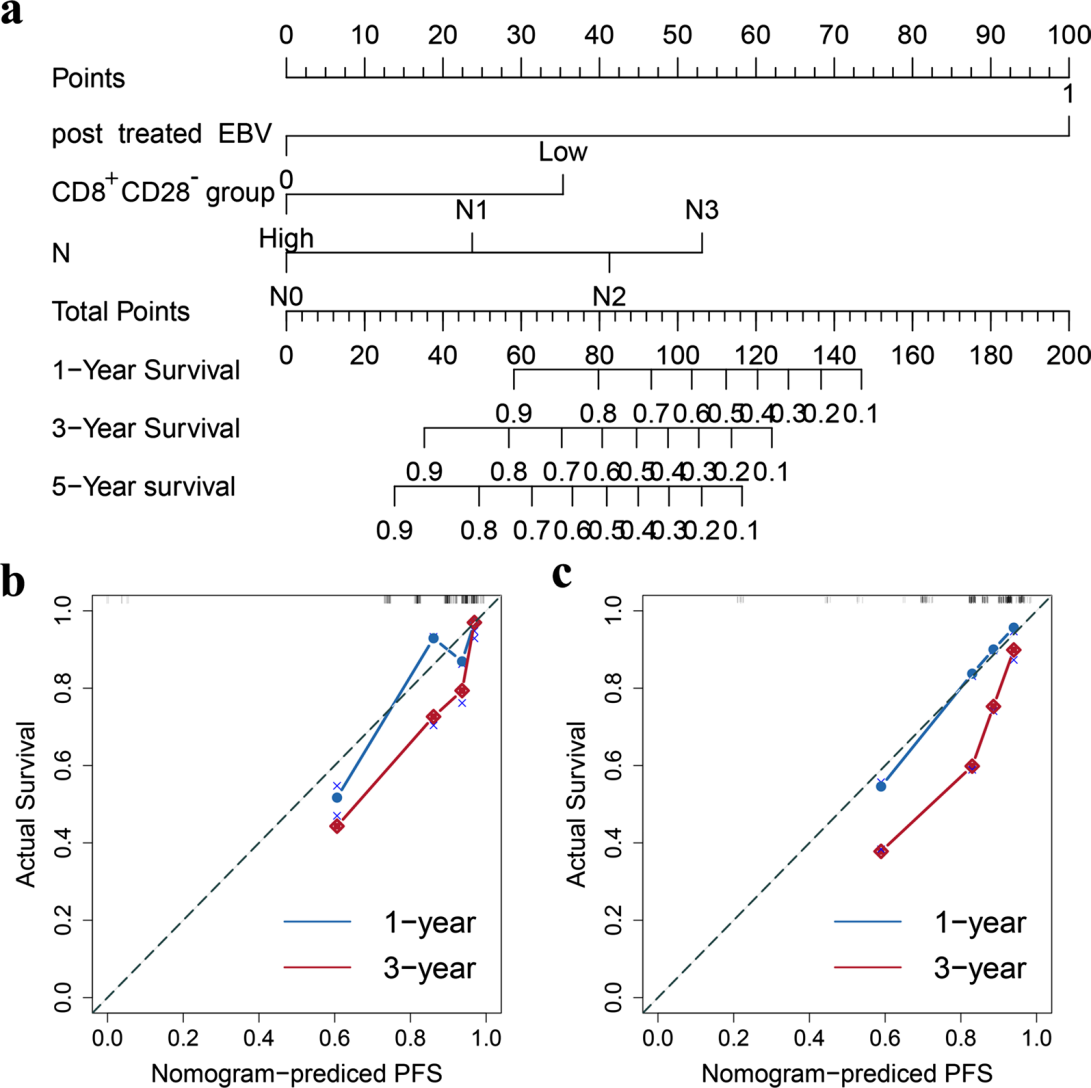
Nomogram and calibration curves for predicting PFS. **a** Nomogram for predicting PFS. **b** One-year and three-year calibration curves in the training cohort. **c** One-year and three-year calibration curve in the validation cohort. In the calibration plot, the X-axis represents the nomogram-predicted probability of survival time, the Y-axis represents the actual survival time, the red lines represents the one-year survival and the blue lines represents the three-year survial

**Fig. 8:**
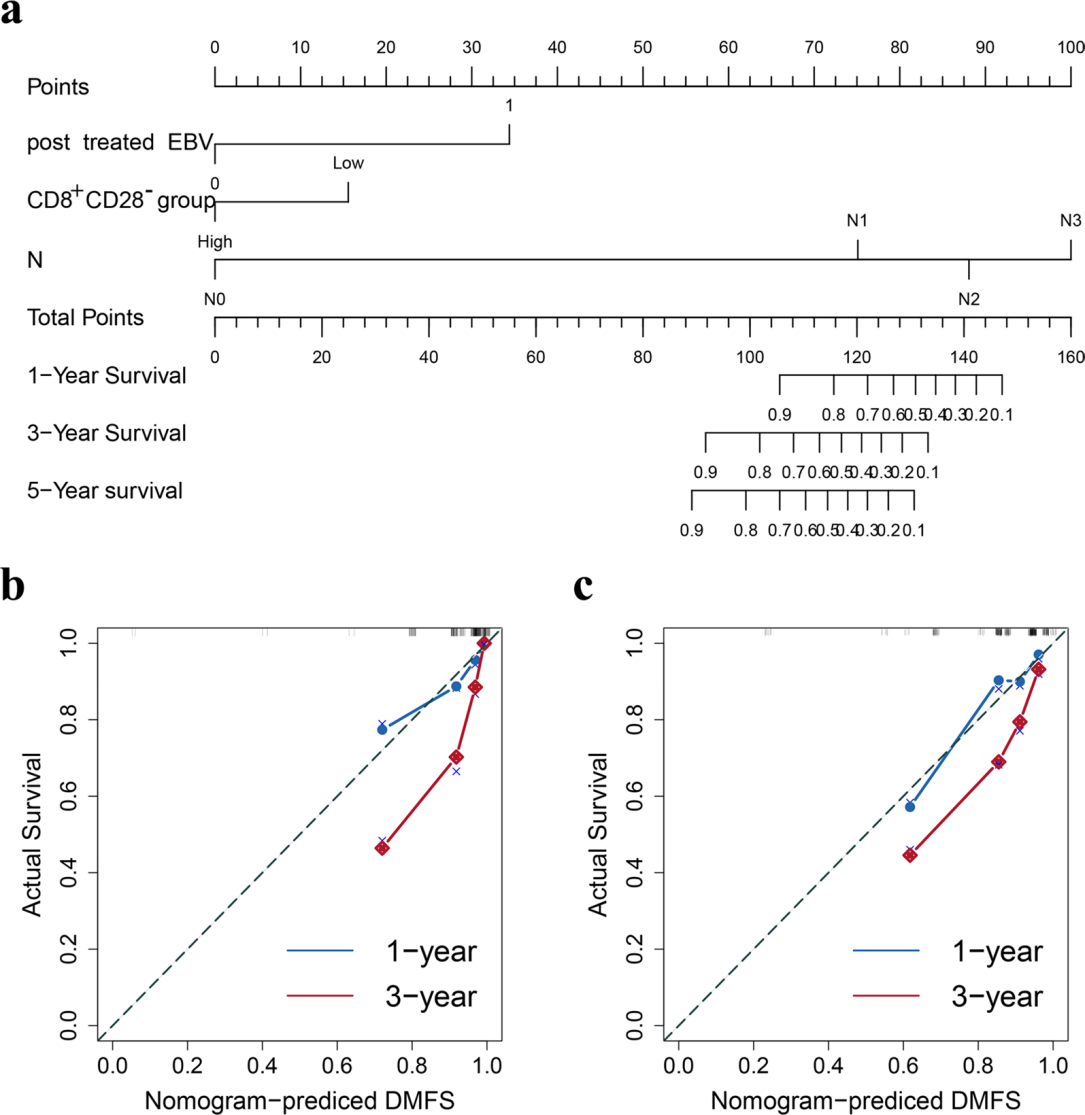
Nomogram and calibration curves for predicting DMFS. **a** Nomogram for predicting DMFS. **b** One-year and three-year calibration curves in the training cohort. **c** One-year and three-year calibration curves in the validation cohort. In the calibration plot, the X-axis represents the nomogram-predicted probability of survival time, the Y-axis represents the actual survival time, the red lines represents the one-year survival and the blue lines represents the three-year survial

We compared the effectiveness of different models, finally, three factors were selected to build a nomogram to predict DMFS: proportion of CD8^+^CD28^−^ T cells, posttreatment EBV and N stage.The nomograms integrating prognostic factors for PFS and DMFS are shown in Fig. 7. For PFS, the nomogram is shown in Fig. 7a. The C-indexes were 0.735 and 0.745 in the training and validation cohorts, respectively (Fig. 7a–c). The calibration curves of the training and validation cohorts (Fig. 7b, c) showed good predictive value for PFS. For DMFS, the nomogram is shown in Fig. 8a. The C-indexes were 0.793 and 0.774 in the training and validation cohorts, respectively, indicating high predictive value. The calibration curves of the training and validation groups (Fig. 8b, c) also verified these results.

## Discussion

NPC is a malignant tumor closely associated with genetic predispositions, EBV infection, occupational hazards, and environmental factors [11, 12]. The incidence of NPC is regionally significant, with most cases occurring in Southeast Asia [13]. The low reactivity and dysfunction of T cells to tumor antigens are the main mechanisms of tumor immune escape, and exhaustion and senescence are the two dominant dysfunctions of T cells [14]. Recently, immune checkpoint inhibitors for cancer immunotherapy have shown benefits for certain types of cancer patients [15, 16]. However, the success rate of immune checkpoint blockade therapies is still low, which suggests that in addition to the exhaustion of T cells, there are another pathway that influences the effects of immunotherapy [17]. Recent studies have shown that T cell immune senescence can reduce vaccine efficacy and increase susceptibility to viral infections and malignant tumors [18, 19]. These studies strongly suggest that T-cell senescence plays an important role in the immune evasion of malignant tumors.

In our study, basic clinical information of patients with NPC, such as EBV, age, sex, lymphocyte subsets and survival time, was collected to determine the prognostic factors closely related to PFS and DMFS through prognostic analysis. Because CD8^+^CD28^−^ T lymphocytes play a role in tumors and inflammation [20], we also evaluated the peripheral blood CD8^+^CD28^−^ T lymphocyte subset by flow cytometry.

In the nomogram for predicting PFS, we included three factors: proportion of CD8^+^CD28^−^ T cells, posttreatment EBV and N stage, which have good predictive value. For the prognostic DMFS nomogram, we included proportion of CD8^+^CD28^−^ T cells, posttreatment EBV, and N stage, and the C-index and calibration curve indicated that this nomogram has good prognostic value.

This study is the first to demonstrate that peripheral blood CD8^+^CD28^−^ T cells can serve as an independent prognostic factor for recurrence and distant metastasis in patients with NPC after radiotherapy, especially in patients with low EBV DNA levels. The proportion of CD8^+^CD28^−^ T cells in peripheral blood can be used as a new immune biomarker for the evaluation of immune function, risk stratification and efficacy prediction in NPC patients.

CD8^+^CD28^−^ T cells are a unique subset of T cells with a nonantigenic specificity regulatory function and are increased in human peripheral blood according to age with declining immune function. NPC is a malignant tumor induced by chronic EBV infection. Both viral infections and tumors may promote the accumulation of CD8^+^CD28^−^ T cells [20]. Some studies have shown that immune-senescence CD8^+^CD28^−^ cells have immunosuppressive functions [21, 22]. Physiological aging, viral infection and autoimmune disease can induce the downregulation of CD28 expression on CD8^+^ T cells [23]. Lower CD28 expression is a marker of T-cell senescence, and increased CD8^+^CD28^−^ subset populations play heterogeneous roles in cancers such as lung cancer and breast cancer [24–26], but few studies have been related to NPC. Our study reported for the first time that senescent peripheral blood CD8^+^CD28^−^ T cells are an independent risk factor for recurrence and distant metastasis in patients with NPC after radiotherapy. As shown in this study, the increase in peripheral CD8^+^CD28^−^ T cells was not associated with the age of NPC patients, which may be due to the combined effect of chronic EBV infection and persistent tumor antigen stimulation. Radiotherapy can further increase the proportion of CD8^+^CD28^−^ T cells in peripheral blood, and the increase lasts for at least 4 months after treatment. Comparing the absolute counts of each subgroup at the end of radiotherapy, the absolute count of CD8^+^CD28^+^ T cells in peripheral blood decreased much more than that of CD8^+^CD28^−^ T cells. These results suggest that CD8^+^CD28^+^ T cells are more insensitive to radiation-induced cell death, and the large proportion of CD8^+^CD28^−^ T cells remaining after radiotherapy may play an important role in immune function remodeling after radiotherapy.

We further explored the immune relationship between CD8^+^CD28^−^ T cells and cytokines. As a costimulator of T cells, CD28 is expressed in all naive T cells in newborns [27]. However, with T-cell activation and differentiation, CD28 expression gradually decreases. The previous study showed that with increasing age and after bone marrow and solid organ transplantation, the CD8^+^CD28^−^ T-cell subset is expressed at higher levels in human peripheral blood, which means that immune function or the immune rejection reaction decreases [28]. Some studies showed that CD8^+^CD28^−^ T cells also have influence on immune regulation. The CD8^+^CD28^−^ T-cell subset can inhibit T-cell activation and proliferation, decrease the secretion of proinflammatory cytokines by activated T cells, and induce activated T-cell apoptosis in vitro [29]. In addition to the stimulation of the TCR by specific antigen epitopes and the removal of inhibitory signals at immune checkpoints, CD28 costimulatory signals are also essential for the functional recovery and continuous activation of T cells [30]. CD28 is a key T-cell costimulatory molecule that binds to the B7 molecule. The binding of CD28 reduces the T-cell receptor signaling threshold required for T-cell activation and provides a qualitatively different signal.

Senescent T cells have phenotypic changes: permanent CD28 expression disappears, cell cycle blockade occurs, and p53, p21, and p16 expression is upregulated [31]. Senescent T cells undergo significant immune function changes, such as defective killing abilities and the development of potent negative regulatory functions that are important for the immune system. In already exhausted CD8 T cells, loss of CD28 expression can also influence the PD-1 immunotherapeutic effect [30]. In our results, peripheral blood CD8^+^CD28^−^ T cells were positively correlated with the inflammatory cytokines IL-1β and IL-5 and the immunosuppressive cytokine IL-10, and the number of CD8^+^CD28^−^ T cells was inversely proportional to CD4/CD8, suggesting that the increase in CD8^+^CD28^−^ T cells may be related to the systemic immunodeficiency of NPC patients. The increase in the number of CD3^+^HLA-DR^+^ activated T cells one month after radiotherapy was significantly lower than that in the group with low levels of CD8^+^CD28^−^ T cells, which is consistent with the immunosuppressive function of CD8^+^CD28^−^ T cells and may suggest that CD8^+^CD28^−^ T cells may play a negative role in immune regulation during the reconstruction of immune function after radiotherapy. Moreover, the proportion of CD8^+^CD28^−^ T cells was associated with prognosis in different stages, which showed that CD8^+^CD28^−^ T cells can be a stratification marker for NPC patients.

Plasma EBV DNA and TNM staging are the main stratification indexes used in the clinic for the prognosis of patients with NPC [29]. Although a high level of plasma EBV DNA can better identify patients with a poor prognosis, other studies and our data show that a high level of plasma EBV DNA pretreatment is associated with disease staging, especially N staging, and is not an independent risk factor for the prognosis of NPC [10, 32]. In addition, our data show that patients with low levels of pretreatment plasma EBV DNA still have a risk of recurrence and distant metastasis. We also explored the prognostic value of the combination of EBV DNA and CD8^+^CD28^−^ T cells. Our results showed that the combination of the two prognostic factors can divide NPC patients into clear survival groups and that NPC patients with higher CD8 + CD28- T cells and EBV > 1500 have a worse prognosis, which can be a new method for predicting NPC prognosis and stratifying NPC patients.

### Limitations

There are some limitations in our study. Although the validation cohort was independent, the data did not come from other medical centers. We will continue our study in different centers. Prospective clinical trials should be designed to further observe the value of the proportion of CD8^+^CD28^−^ T cells in peripheral blood before treatment for treatment regimen screening and efficacy prediction, especially for immunotherapy.

## Conclusions

We screened and identified the proportion of CD8^+^CD28^−^ T lymphocytes in peripheral blood before radiotherapy as an important prognostic factor for NPC metastasis. The model for predict DMFS and PFS we built has good predictive power.

## Supplementary Information

**Additional file 1.** Raw data of NPC patients.

**Additional file 2.** Follow-up raw data.

**Additional file 3.** NPC patients characteristics.

**Additional file 4.** Univariate Cox regression analysis results.

**Additional file 5.** Multivariate Cox regression analysis results.

**Additional file 6.** Raw data of healthy controls.

**Additional file 7.** Raw data of cytokine detection.

**Additional file 8.** KM plots for CD28^+^CD28^-^ T-cell and EBV. a Kaplan-Meier plot for the proportion of CD8^+^CD28^-^ T cells for PFS in the training cohort. b. Kaplan-Meier plot for the proportion of CD8^+^CD28^-^ T cells for PFS in the Validation cohort. The blue represents low proportion of CD8^+^CD28^-^ T cells and red line represents high proportion of CD8^+^CD28^-^ T cells.

**Additional file 9.** KM plot for different stages. Kaplan-Meier plot for the different stages for DMFS. The blue line represents stage I/II, the yellow line represents stage III and the red line represents stage IV.

## Data Availability

The datasets used and/or analyzed during the current study are available from the corresponding author on reasonable request.

## Abbreviations

PFS: Progression-free survival
DMFS: Distant metastasis-free survival
IMRT: Intensity-modulated radiotherapy
CCRT: Concurrent chemoradiotherapy
NPC: Nasopharyngeal carcinoma
ECOG: Eastern cooperative oncology group
uniCox: Univariate cox
multiCox: Multivariate cox
LASSO: Least absolute shrinkage and selection operator
EBV: Epstein‒Barr virus
NEU: Neutrophils
LY: Lymphocytes
