# Supplementary figures and images for: High proportion of circulating CD8 + CD28- senescent T cells is an independent predictor of distant metastasis in nasopharyngeal canrcinoma after radiotherapy
Source: J Transl Med. 2023 Jan 31;21:64. doi: 10.1186/s12967-023-03912-2 (PMC9887944; doi:10.1186/s12967-023-03912-2)

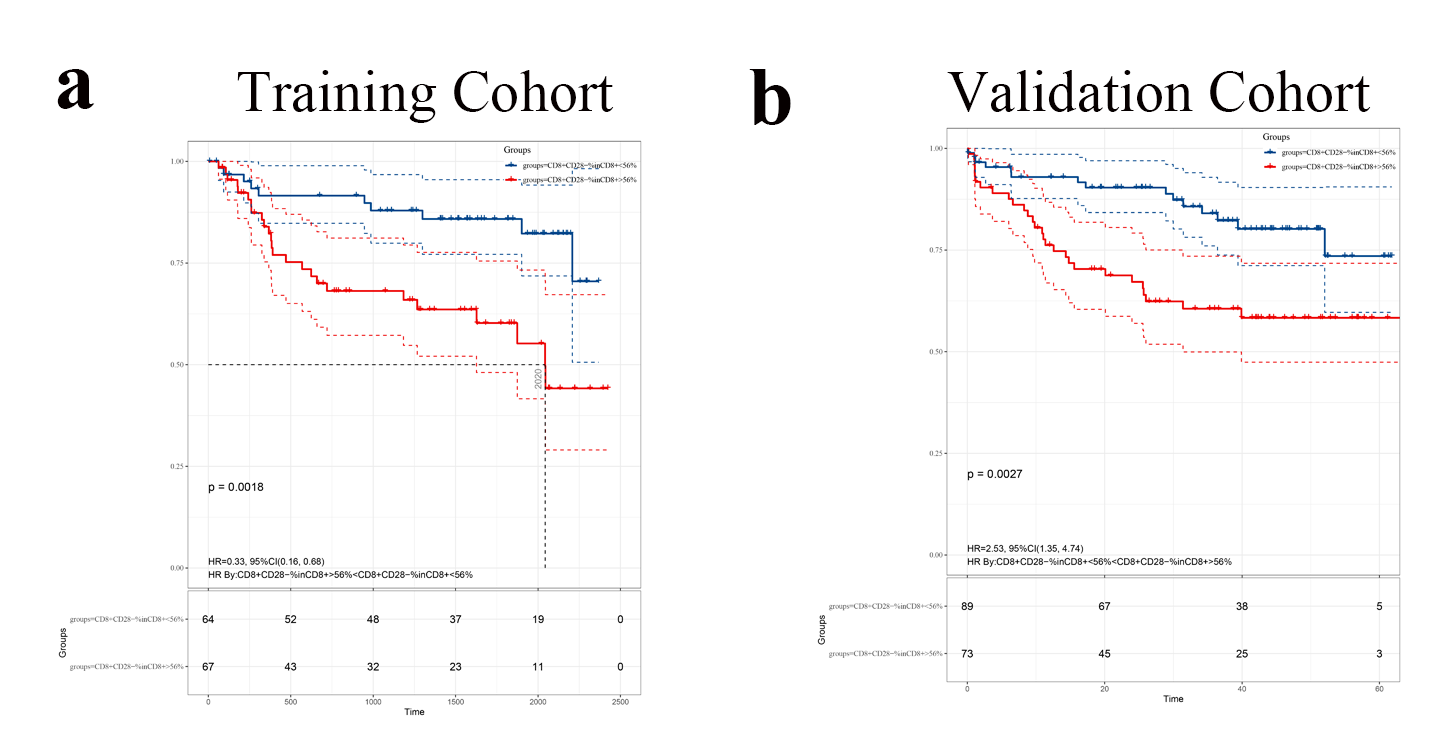

Supplement: Supplementary file 8 — Additional file 8. KM plots for CD28+CD28- T-cell and EBV. a Kaplan-Meier plot for the proportion of CD8+CD28- T cells for PFS in the training cohort. b. Kaplan-Meier plot for the proportion of CD8+CD28- T cells for PFS in the Validation cohort. The blue represents low proportion of CD8+CD28- T cells and red line represents high proportion of CD8+CD28- T cells. [file 12967_2023_3912_MOESM8_ESM.tif]

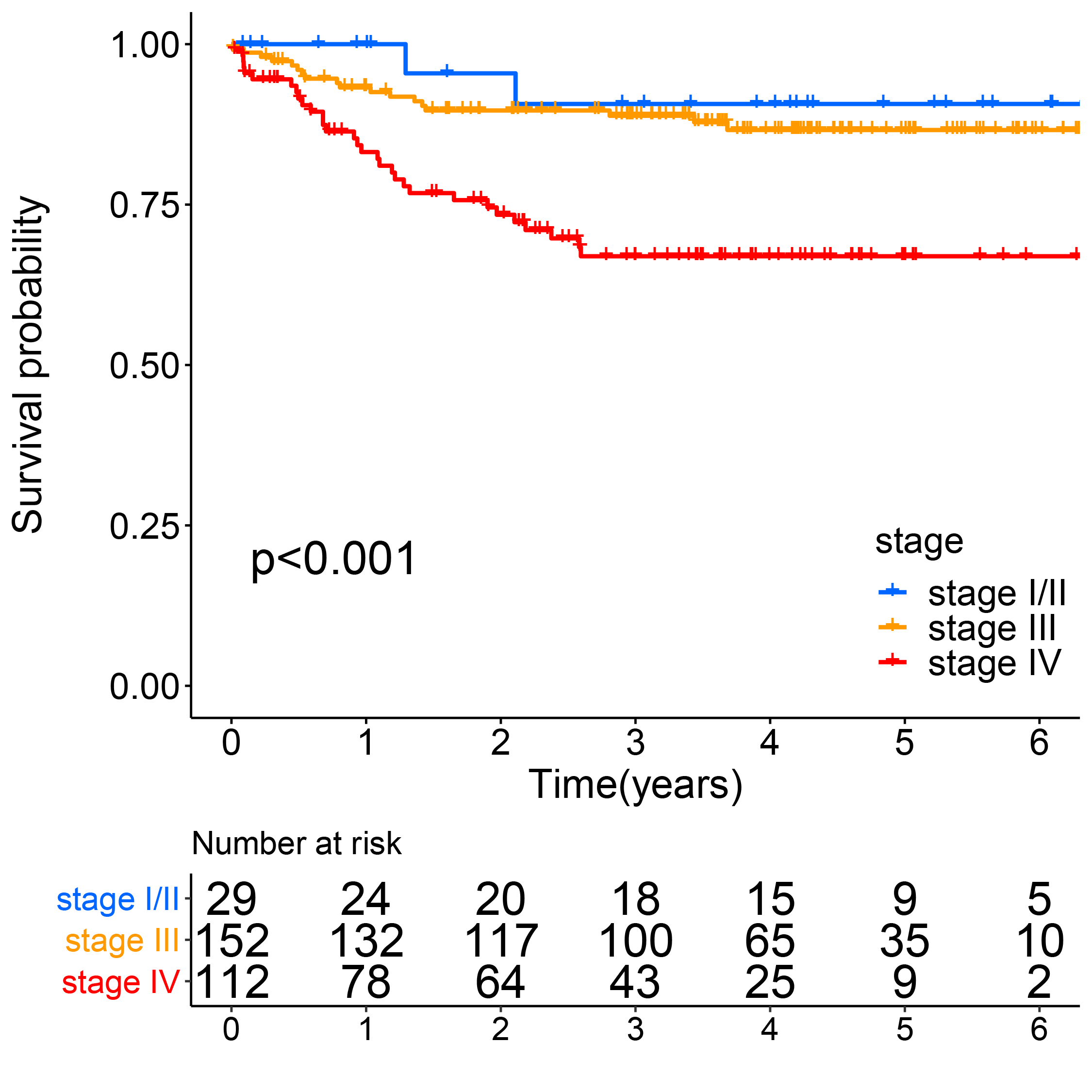

Supplement: Supplementary file 9 — Additional file 9. KM plot for different stages. Kaplan-Meier plot for the different stages for DMFS. The blue line represents stage I/II, the yellow line represents stage III and the red line represents stage IV. [file 12967_2023_3912_MOESM9_ESM.tif]
